# The Brain Metabolome Is Modified by Obesity in a Sex-Dependent Manner

**DOI:** 10.3390/ijms25063475

**Published:** 2024-03-20

**Authors:** Jennifer E. Norman, Dragan Milenkovic, Saivageethi Nuthikattu, Amparo C. Villablanca

**Affiliations:** 1 Division of Cardiovascular Medicine, Department of Internal Medicine, University of California, Davis, 1 Shields Ave, Davis, CA 95616, USA; snuthikattu@ucdavis.edu (S.N.); avillablanca@ucdavis.edu (A.C.V.); 2 Department of Nutrition, University of California, Davis, 1 Shields Ave, Davis, CA 95616, USA; dmilenkovic@ucdavis.edu

**Keywords:** obesity, brain, metabolomics, sex differences, cognitive function

## Abstract

Obesity is linked to cognitive decline and metabolic dysregulation in the brain, yet the role of sex is relatively unexplored. We sought to explore the effects of obesity and sex on the brain metabolome. In male and female *ob/ob* and wild-type mice, we assessed whole brain untargeted metabolomics by liquid chromatography–mass spectrometry, behavior by open field test, and cognitive function by Y-maze and Morris water maze. The metabolic profiles of *ob/ob* and wild-type mice differed in both sexes. There were more obesity-altered brain metabolites in males than females. Thirty-nine metabolites were unique to males, 15 were unique to females, and five were common to both sexes. Two of the common metabolites were involved in nicotinamide adenine dinucleotide homeostasis. A key feature of the metabolites identified in males was an increase in free fatty acids. In females, a unique feature was the presence of the neuro-modulatory metabolites 2-linoleoyl glycerol and taurine. The behavioral effects of obesity were only seen in females. These results demonstrate that most impacts of obesity on the brain metabolomic profile are sex-specific. Our work has implications for understanding the role of obesity in brain metabolism and the differential contribution of obesity to cognitive decline in males and females.

## 1. Introduction

Obesity, defined as a body mass index (BMI) of ≥30 kg/m^2^, is a major public health concern, contributing to increased morbidity and mortality [1]. Obesity during mid-life is associated with an increased risk of developing dementia later in life [2,3,4], independent of other comorbidities [5]. Gray matter is reduced in obesity in multiple brain regions, including the hippocampus, which is a region important in memory and affected by Alzheimer’s disease [6]. However, the connection between obesity and dementia is controversial, as there are many comorbidities of obesity that also increase the risk of developing dementia, such as insulin resistance, diabetes, hypertension, and cardiovascular disease [6,7]. Further, in a phenomenon known as the “obesity paradox”, the association between obesity and cognitive decline is not apparent over the age of 60, as it is in younger individuals [6,8]. Thus, obesity impacts the brain and the risk for cognitive decline, but the relationship has not been fully elucidated, and more research is needed.

Obesity is associated with metabolic dysregulation, and the brain is not an exception to this. Altered basal brain metabolism occurs with obesity, as previously demonstrated in a mini-pig model [9]. Altered brain glucose metabolism in persons with obesity is a proposed potential mechanism for the increased risk of cognitive decline [10]. Additionally, persons with obesity have greater brain fatty acid uptake when compared to lean controls [11]. Alterations in brain metabolism and mitochondrial function impact cognitive processes and may be linked to various neurological disorders [12]. Thus, studying the brain metabolome may provide insights into the effects of obesity on the brain and the potential implications for cognition.

Sex has been recognized as an important, yet often overlooked, modifier of biological phenomena. Sex differences were reported in adipose tissue, including its distribution, gene expression, metabolism, and inflammation; the presentation of obesity; the risk of obesity-associated metabolic diseases; and the effects of obesity on the sympathetic nervous system [13,14,15]. Additionally, sex differences were found in cognition, as well as the presentation and incidence of neurological diseases [16]. For example, men were reported to have better spatial ability, while women were shown to have greater verbal ability and perceptual speed [17]. It was noted that sex differences may be due to differences in strategy and outcome preference rather than ability [18]. Therefore, it appears that sex differences in cognition may be related to variations in cognitive tasks rather than domain differences. During the progression of Alzheimer’s disease, sex differences were found in the peripheral metabolome [19]. Sex differences have also been described in brain metabolism, including glycolysis and mitochondrial metabolism [20,21]. Further, there are sex differences in the relationship between obesity and the brain metabolic rate [22]. Therefore, it is plausible that obesity exerts sex-specific impacts on the brain metabolomic profile, yet to our knowledge, this was not previously investigated.

In the current study, we sought to determine the impact of obesity on the brain metabolome and whether it is modulated by biological sex. To investigate this, we used the well-characterized *ob/ob* mouse model. Due to a homozygous mutation, *ob/ob* mice lack leptin, a hormone important in the regulation of body weight, and become obese as early as four weeks of age [23,24]. We assessed the brain metabolome using untargeted metabolomics as measured by liquid chromatography–mass spectrometry (LC-MS). Additionally, we assessed the behavior and cognitive function of these mice. We hypothesized that obesity would modify the brain metabolome and that these modifications would be characterized by increased lipids, particularly fatty acids. Further, we hypothesized that there may be a strong interaction of sex with obesity and the brain metabolome, perhaps characterized by a greater detrimental impact of obesity in one sex as compared to the other.

## 2. Results

### 2.1. Obesity Alters the Brain Metabolome in a Sex-Specific Manner

**Figure 1:**
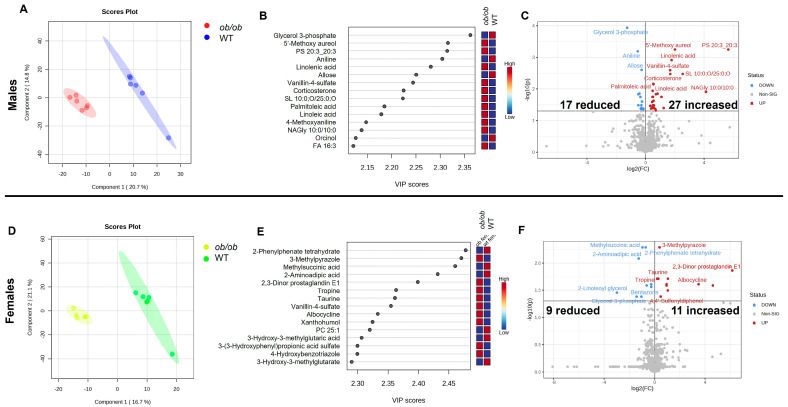
Obesity modifies the brain metabolome in male and female mice, with a greater number of metabolites altered in males. Partial least squares-discriminate analyses (PLS-DA) plots of brain metabolic profiles in *ob/ob* and wild-type (WT) male (**A**) and female (**D**) mice. Small circles represent individual mouse brain samples, while the larger shaded region of the same color represents the 95% confidence interval of the group (in panels (**A**,**D**)). Variable importance projection (VIP) scores of the top 15 variables driving the separation between *ob/ob* and WT mice for component 1 for males (**B**) and females (**E**). Volcano plot of brain metabolites differing between *ob/ob* and WT mice for males (**C**) and females (**F**). No fold change cutoff was applied. All metabolites denoted in blue (decreased) and red (increased) in panels **C** and **F** were significant as determined by adjusted *p* < 0.05.

We first conducted global analyses of the untargeted metabolomics data for males and females, separately. When the data were assessed using partial least squares-discriminate analyses (PLS-DA), the metabolic profile of *ob/ob* mice was shown to differ from wild-type (WT) mice in both males and females (Figure 1, panels A and D, respectively). The top 15 metabolites that contribute to the clustering of the metabolic profiles in the PLS-DA, as determined by variable importance projection (VIP) score, are shown for males and females in Figure 1, panels B and E, respectively. Only one of the top 15 metabolites, vanillin-4-sulfate, drove the separation between *ob/ob* and WT in both males and females. In males, the top three metabolites (as determined by VIP score) which drove the separation between *ob/ob* and WT mice were glycerol-3-phosphate, 5′-methozy aureol, and PS (20:3_20:3). Additionally, four of the top 15 metabolites were fatty acids (linolenic acid, palmitoleic acid, linoleic acid, and FA 16:3); each of these fatty acids was detected in higher quantities in the brain of *ob/ob* males when compared to WT. In females, the top three metabolites driving the separation between WT and *ob/ob* mice were 2-phenylphenate tetrahydrate, 3-methylpyrazole, and methylsuccinic acid.

**Figure 2:**
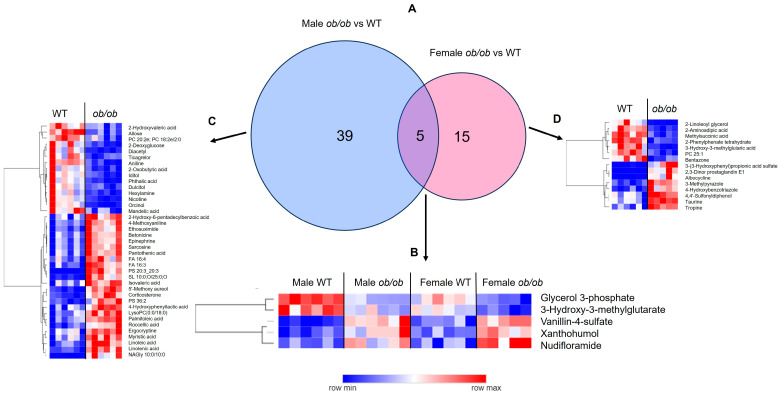
The majority of metabolites altered by obesity are sex-dependent. (**A**) Venn diagram comparison of metabolites significantly different by genotype (*ob/ob* vs. wild-type (WT)) between males and females. Heatmaps of metabolites common to males and females (**B**), specific to males (**C**), and specific to females (**D**). In all heatmaps, dark blue represents lower levels, dark red represents higher levels, and lighter shades indicate intermediate levels of metabolites, as indicated in the color bar.

Next, we used pairwise comparison to determine which metabolites differed between the brains of *ob/ob* and WT mice for each sex separately. In males, 44 metabolites differed between *ob/ob* and WT mice, 27 of which were increased and 17 were reduced in *ob/ob* as compared to WT (Figure 1C). In contrast, in females, 20 metabolites differed between *ob/ob* and WT mice, 11 of which were increased and nine were reduced in *ob/ob* as compared to WT mice (Figure 1F). A comparison of the metabolites altered by obesity in males and females demonstrated that five metabolites were common to both sexes, 39 were unique to males, and 15 were unique to females (Figure 2A). The five common metabolites in males and females were glycerol-3 phosphate, 3-hydroxy-3-methylglutarate, vanillin-4-sulfate, xanthohumol, and nudifloramide; these five metabolites were altered by obesity in a similar manner in both sexes (Figure 2B). Heatmaps of the metabolites altered by obesity, which were unique to males and females, are shown in Figure 2, panels C and D, respectively. The complete lists of metabolites differing between *ob/ob* and WT mice can be found in Tables S2 and S3 (for males and females, respectively).

### 2.2. Lipids Are the Predominant Metabolites Altered by Obesity in the Brain

**Figure 3:**
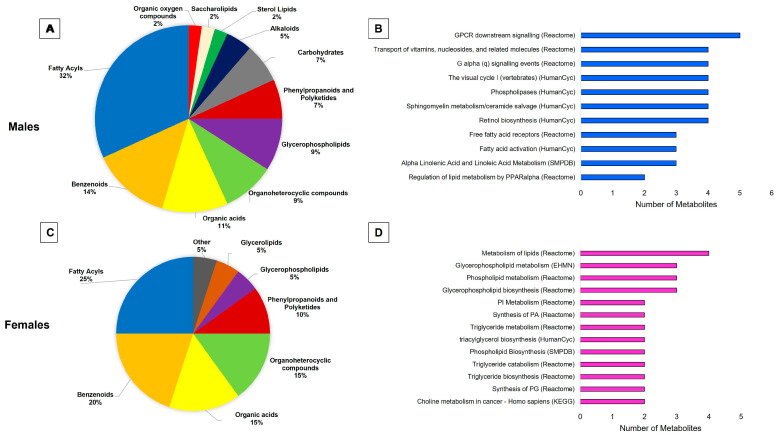
Categorization and pathway analysis of significant metabolites demonstrate a strong effect of obesity on brain lipid metabolism. Pie charts depicting the classification of significantly differing metabolites in males (**A**) and females (**C**). Pathways over-represented in significant metabolites altered by obesity in males (**B**) and females (**D**). Pathways shown were selected based on having a Q value less than 0.1.

Categorization and pathway analysis further explored the metabolites which differed in the brains of *ob/ob* and WT male and female mice. The categorization of the metabolites can be found in Figure 3, panel A (males) and panel C (females). For both sexes, fatty acyls were the predominant category of metabolites altered by obesity, making up a larger proportion of the altered metabolites in males (32%) than in females (25%). Comparing males to females, a smaller portion of altered metabolites were benzenoids (14% vs. 20%), organic acids (11% vs. 15%), and organoheterocyclic compounds (9% vs. 15%). In contrast, glycerophospholipids made up a larger proportion of the metabolites altered in males as compared to females (9% vs. 5%). The results of the pathway analyses can be seen in Figure 3, panel B (males) and panel D (females). Although there were no common significantly over-represented pathways between males and females, both sexes had altered metabolites from lipid-related pathways. Males showed an over-representation of G-protein coupled receptor (GPCR) signaling and fatty acid metabolism-related pathways. On the other hand, females showed an over-representation of glycerophospholipid and triglyceride metabolism-related pathways.

### 2.3. Global Comparison Using PLS-DA and Hierarchical Clustering Differentiate the Effects of Obesity and Sex

**Figure 4:**
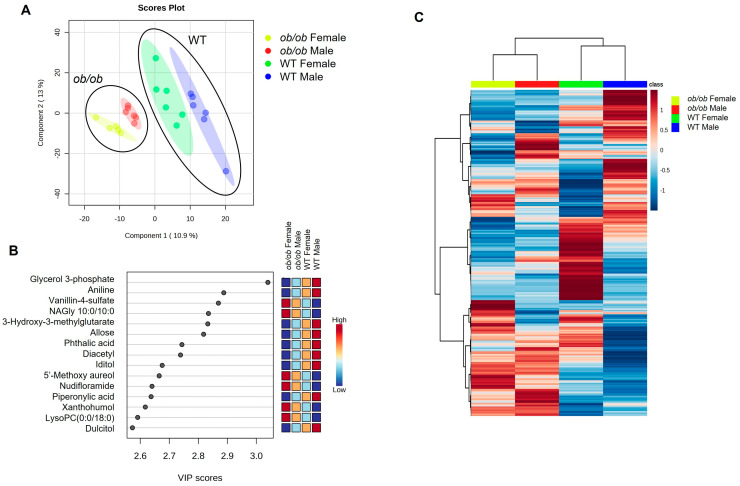
Obesity has a stronger effect on the brain metabolome than biological sex, as shown by partial least squares-discriminate analyses (PLS-DA) and hierarchical clustering. (**A**) PLS-DA of *ob/ob* and wild-type (WT) male and female brain metabolic profiles. Small circles represent individual mouse brain samples, while the larger shaded region of the same color represents the 95% confidence interval of the group. Black circles group samples of the same genotype for ease of visualization. (**B**) Variable importance projection (VIP) scores of top 15 variables for component 1. (**C**) Hierarchical clustering heatmap of group averages of *ob/ob* and WT male and female metabolites. Dark blue represents lower levels, dark red represents higher levels, and lighter shades indicate intermediate levels of metabolites, as indicated in the color bar.

Next, using PLS-DA and hierarchical clustering, we compared the global metabolic profile of all four study groups together (female *ob/ob*, male *ob/ob*, female WT, and male WT). Regardless of sex, the global metabolic profile of *ob/ob* mice clustered separately from that of WT mice in the PLS-DA plot; males and females clustered separately within each genotype (Figure 4A). The top 15 metabolites contributing to the PLS-DA clustering are listed (Figure 4B), with glycerol 3-phosphate emerging as the top metabolite driving group separation (as measured by VIP score). Hierarchical clustering analysis also demonstrated that the global metabolic profile of *ob/ob* males and females differed from that of WT males and females (Figure 4C). Thus, both sex and obesity contributed to differences in the brain metabolic profile, with the effect of obesity being stronger in driving group separation than biological sex.

### 2.4. Obesity Alters Peripheral Metabolic Parameters, Locomotion, and Behavior

Both male and female *ob/ob* mice exhibited significantly (*p* < 0.05) higher body weight, were hyperinsulinemic and hypercholesterolemic, and showed impaired glucose tolerance as compared to WT mice of the same sex. Female *ob/ob* mice had elevated fasting glucose levels compared to sex-matched WT mice, but no significant difference was seen in males. A summary of metabolic parameters for *ob/ob* and WT mice can be found in Table 1.

**Figure 5:**
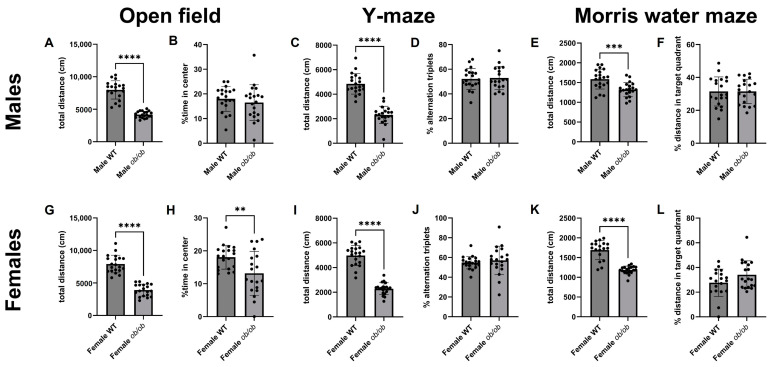
Obesity reduces locomotion in both sexes and alters behavior in female mice. Total distance traveled during the open field test is shown for males (**A**) and females (**G**). Percentage of time spent in the center of the open field is shown for males (**B**) and females (**H**). Distance traveled in the Y-maze is shown for males (**C**) and females (**I**). Y-maze performance, as measured by % alternation triplets, is shown for males (**D**) and females (**J**). Total distance traveled in the Morris water maze is shown for males (**E**) and females (**K**). Morris water maze performance, as assessed by percentage of distance in the target quadrant, is shown for males (**F**) and females (**L**). ** *p* < 0.01; *** *p* < 0.001; **** *p* < 0.0001.

We also assessed behavior and cognition to pair with the brain metabolic data (Figure 5). In the open field test, Y-maze, and Morris water maze (MWM), *ob/ob* mice travelled less distance as compared to WT mice, regardless of sex. In addition, female mice, but not male mice, spent significantly (*p* < 0.05) less time in the center of the open field test (Figure 5, panels B and H).

### 2.5. Obesity-Induced Brain Metabolic Changes Correlated with Peripheral Metabolic Characteristics, Behavior, and Cognitive Endpoints

We utilized correlation analyses to investigate the relationship between the brain metabolites altered by obesity and phenotypic measures of peripheral metabolic characteristics (fasted serum glucose, insulin, total cholesterol, and performance on the glucose tolerance test (GTT)) and behavior/cognitive function (distance traveled and performance on the open field, Y-maze, and MWM tests). Correlations were performed separately for males and females using the obesity-induced delta, as defined by the difference between the *ob/ob* mice and the average of sex-matched WT mice.

**Figure 6:**
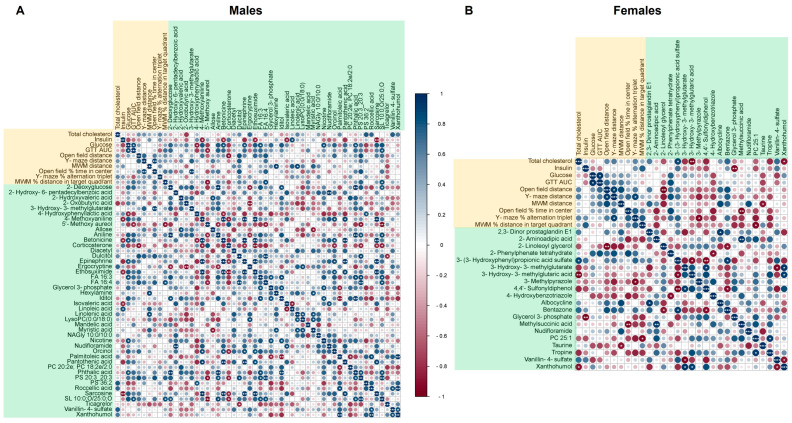
Correlation of brain metabolites with peripheral metabolic characteristics and behavioral/cognitive performance. Pearson correlation matrices for the obesity-induced deltas (difference between *ob/ob* mice and WT average of the same sex) of significant metabolites, peripheral metabolic characteristics, behavioral outcomes, and cognitive outcomes for males (**A**) and females (**B**). Phenotypic variables are highlighted in orange, and metabolites are highlighted in green. The color of circle indicates the Pearson’s r value from dark red representing −1 to dark blue representing 1. The number of white asterisks within the colored circles represents the *p*-value as follows: * *p* < 0.05, ** *p* < 0.01, and *** *p* < 0.001.

In males, there were 21 significant correlations between the 44 obesity-altered metabolites and phenotypic measures (Figure 6A). In contrast, females had nine significant correlations between the 20 obesity-altered metabolites and phenotypic measures (Figure 6B). Of the metabolites altered by obesity common to males and females, the relationships to peripheral metabolic characteristics and behavior/cognition were different for each of the sexes. For example, xanthohumol was negatively correlated with total cholesterol in females, not in males. Glycerol 3-phosphate was negatively correlated with serum insulin in females, not in males. In contrast, 3-hydroxy-3-methylglutarate was positively correlated with the percentage of time spent in the center of the open field in males, not in females. 

When focusing on brain metabolite relationships with behavior and cognitive function, a few significant correlations were found. The percent of time spent in the center of the open field was positively correlated with 3-hydroxy-3-methylglutarate in males and negatively correlated with methylsuccinic acid in females. The percentage of alternation triplets in the Y-maze did not have any significant relationship to altered brain metabolites in males, but negatively correlated with 3-methylpyrazole in females. The percent of time spent in the target quadrant on the MWM negatively correlated with 5′-methoxy aureol and positively correlated with FA 16:4 and SL10:0;O/25:0;O in males. On the other hand, the percent of time spent in the target quadrant in females negatively correlated with PC25:1. Lists of the significant correlations between obesity-altered metabolites and phenotypic measures of peripheral metabolic characteristics, behavior, and cognitive function for males and females are provided in Tables S4 and S5, respectively.

## 3. Discussion

This study addressed the impact of obesity and biological sex on the brain metabolome, using the *ob/ob* mouse model of obesity, and examined the behavioral, cognitive, and peripheral metabolic correlates. The phenotypic characteristics of the *ob/ob* model on the C57Bl/6J background in our study were consistent with prior reports [24]. We found differences between genotype (*ob/ob* vs. WT) and sex (male vs. female) in the global brain metabolic profile. While the effect of obesity appeared to be greater than that of sex, there was an effect of sex on the response of the brain metabolic profile to obesity. Most effects of obesity on brain metabolites were sex-specific; however, a few were sex-independent. Further, obesity impacted behavior in female mice and locomotion in both sexes. We discuss the *ob/ob* mouse as a model for human obesity, our findings in the context of sex-specific effects of obesity on the brain metabolic profile, the sex-independent effects, potential implications for dementia, and how our data fit with the existing literature on brain metabolism from murine models with obesity.

### 3.1. The ob/ob Mouse as a Model of Human Obesity

Obesity is a growing global problem associated with comorbidities affecting most systems in the body, including hypertension, cardiovascular diseases, respiratory complications, stroke, type 2 diabetes mellitus, dyslipidemia, cancers, and non-alcoholic fatty liver disease [1,25]. Similarly to humans, obesity in *ob/ob* mice is accompanied by impaired glucose tolerance, hyperinsulinemia, and hyperlipidemia [1,24,25]. However, it should be noted that there are some differences in presentation between *ob/ob* mice and humans; for example, the increase in lipids in *ob/ob* mice is primarily seen in high-density lipoproteins (HDL) rather than very low-density lipoproteins (VLDL) or low-density lipoproteins (LDL) [24]. Although data on sex differences in the *ob/ob* murine model are scarce due to the lack of inclusion of females in studies, some described sex differences seem to parallel those found in humans. For example, female *ob/ob* mice were shown to have elevated blood pressure compared to WT counterparts, while males did not [26]. Similarly, in humans, studies demonstrated that hypertension is more strongly associated with obesity in women than in men [27].

It is important to acknowledge that there are murine models of obesity other than the *ob/ob* model, each with its own strengths and weaknesses. Some of the other well-known genetic murine obesity models include the *db/db* (leptin receptor-deficient) and the lethal yellow agouti model (ectopic ubiquitous expression of the agouti protein). We chose to utilize the *ob/ob* model over the other models as we felt it had advantages to address our research question. While the *ob/ob* model does have some phenotypic aspects of type 2 diabetes mellitus (T2DM), such as glucose intolerance and insulin resistance, the *db/db* model exhibits a more severe glucose intolerance and T2DM-like phenotype than the *ob/ob* model [24,28]. We wanted to focus on the effects of obesity itself, rather than T2DM, and thus, opted for the model with a less severe T2DM phenotype. Meanwhile, the lethal yellow agouti has a later onset of obesity and is prone to tumors [24]. A non-genetic murine model of obesity that is also widely used is the high-fat diet (HFD)-induced obesity. While this is also an important model of obesity, we chose not to use this model in our study as the increased dietary fat would undoubtedly affect the brain metabolome, in addition to obesity itself. Thus, the *ob/ob* model was best suited for our research question on the effects of obesity on the brain metabolome.

### 3.2. Sex-Specific Effects of Obesity on the Brain Metabolic Profile

This study demonstrated that sex is an important modulator of the brain’s metabolic response to obesity. Most of the brain metabolites altered by obesity were unique to one of the sexes, with obese males having more altered metabolites than obese females. Additionally, for metabolites altered by obesity in both sexes, the relationships to phenotypic measures were different in males and females.

Pathway analyses indicated that GPCR and free fatty acid metabolism were over-represented in males. GPCRs were implicated as treatment targets in neurodegenerative disorders and cognition [29]. In the current study, of the metabolites with the top 15 VIP scores driving the separation between obese and control male mice, four were free fatty acids. A total of 11 free fatty acids were significantly altered by obesity in males, including two hydroxy fatty acids, one branched fatty acid, and one oxo fatty acid. All seven straight-chain unmodified fatty acids and the branched-chain fatty acid were increased in the obese state. These findings are in accordance with a previous study that showed an increase in fatty acid uptake by the brain in obesity [11]. Further, brain fatty acid levels are altered in Alzheimer’s disease [30]. Thus, the changes we observed in males are supported by previous studies and indicate a potential mechanism for the increased risk of cognitive decline seen with obesity, mediated by increased brain fatty acids.

In females, lipid metabolism, including glycerophospholipid and triglyceride metabolism, was over-represented in the metabolites altered by obesity. Two of the female-specific metabolites altered by obesity, which mapped to the over-represented pathways, have neuro-modulatory functions: 2-linoleoyl glycerol and taurine. 2-Linoleoyl glycerol was decreased in obese females in our study and is a monoacylglycerol, which is a partial agonist of the cannabinoid type 1 receptor (CB1) [31]. Interestingly, CB1 agonists can reduce excitotoxicity caused by excessive excitatory signaling [32]. This suggests that obesity could increase excitotoxicity through reduced 2-lineoyl glycerol. Taurine, which was increased in the brains of obese female mice, has inhibitory effects on γ-Aminobutyric acid, glycine, and N-methyl-D-aspartate receptors [33,34,35,36,37,38,39]. Interestingly, our previous work also demonstrated a reduction of 2-linoleyl glycerol and an increase in taurine in the brain as a female-specific response to diabetes [40]. Thus, obesity-induced changes in the brain metabolome in females may alter neural signaling and contribute to cognitive decline.

Although beyond the scope of the current study, potential mechanisms behind the observed sexual dimorphism include direct effects of sex hormones, gonadal sex, and genetic effects of sex chromosomes. Peripheral metabolism, brain structure, and behavior are known to be impacted by both sex hormones and sex chromosomes [41,42,43,44]. Brain regional differences in both grey matter volume (GMV) and sex chromosome gene expression were described, and they correlate with one another [45]. For example, females were shown to have greater GMV than males in the prefrontal cortex, while males were shown to have greater GMV than females in the ventral temporal and occipital regions [45]. However, given that our study utilized whole brain tissue for metabolomics analyses, we cannot draw conclusions as to whether the observed sex differences are due to differences in specific regions or are widespread. Therefore, it is likely that a few, or even all, of these factors combine to influence the brain metabolome. The contributions of hormones, gonadal sex, and chromosomal sex to the brain metabolic response to obesity and whether these differences are regional or widespread remains an area for further investigation.

### 3.3. Sex-Independent Effects of Obesity on the Brain Metabolome

Two of the five metabolites which were altered in both males and females, glycerol 3-phosphate and nudifloramide, are involved in nicotinamide adenine dinucleotide (NAD+) homeostasis, a key process in energy production. Glycerol 3-phosphate was the metabolite with the highest VIP score when PLS-DA analysis was conducted on all four study groups. Additionally, glycerol 3-phosphate was reduced by obesity to a level less than 50% of WT controls in both males and females. The glycerol 3-phosphate shuttle is a system which transports reducing equivalents from reduced nicotinamide adenine dinucleotide (NADH) into the mitochondria by interconverting dihydroxyacetone phosphate and glycerol 3-phosphate, in order to regenerate cytosolic NAD+ [46]. This system is important in neurons for metabolic flexibility and is activated when energy demands increase, such as during long-term potentiation [47,48]. Nudifloramide was also in the top 15 metabolites by VIP score in the PLS-DA analysis of all four study groups, and levels in the brain were increased by obesity in both sexes. Nudifloramide, also known as N-methyl-2-pyridone-5- carboxamide, is one of the end products of NAD+ degradation, and its accumulation was shown to have toxic properties [49]. NAD+ levels decrease with age, which may occur through increased degradation, and were linked to cognitive decline [50,51,52]. Nicotinamide N-methyltransferase (NNMT) is a key enzyme in the pathway which generates nudifloramide during NAD+ degradation [51]. The expression of NNMT was shown to be increased in obese adipose tissue, and its inhibition was beneficial for weight loss [53,54]. Further, its expression in neurons is increased in the brains of individuals with Alzheimer’s disease [55]. Therefore, altered NAD+ homeostasis within the brain may be an additional pathway to explain the connection between obesity and cognitive decline both in males and in females.

### 3.4. Potential Implications for Cognitive Decline

In addition to the observed obesity-induced shift in the brain metabolome, we found differences in locomotion and behavior in obese mice. Regardless of sex, obese mice traveled less distance than controls on all tests conducted. Others also observed reduced distance traveled in the open field test [56]. One potential explanation is that reduced locomotion may be due to physical restriction caused by the larger size and weight of the *ob/ob* mice. A reduction in the percent of time spent in the center of the open field test, which is behavior indicative of anxiety [57], was seen in females but not males in our study. Current knowledge on the connection between anxiety and obesity is inconclusive [58], but our results indicate that sex may be a modifier of that relationship. Although our study did not find any effects of obesity on cognitive function and memory as assessed through the Y-maze and the MWM during the study period, the observed shift in the brain metabolic profile may contribute to cognitive changes at later time points and is an area for further study.

Our data showed significant correlations between obesity-altered brain metabolites and the behavioral and cognitive endpoints. In males, the percent of time spent in the center of the open field was positively correlated with 3-hydroxy-3-methylglutarate. 3-Hydroxy-3-methylglutarate, also known as β-hydroxy-β-methylglutarate (HMG), can be released when HMG complexed to coenzyme A (HMG-CoA) builds up in the mitochondria. HMG-CoA is an intermediate in the mevalonate pathway, which leads to the production of cholesterol. The production of cholesterol in the brain, which is important for myelin sheaths and neuronal membranes, is separated from the periphery by the blood–brain barrier [59]. Thus, the alteration in 3-hydroxy3-methylgutarate, and its correlation with time spent in the center of the open field may indicate an interaction between obesity, brain cholesterol metabolism, and anxiety-like behavior in males. The percent of time spent in the target quadrant on the MWM negatively correlated with 5′-methoxy aureol and positively correlated with FA 16:4 and SL10:0;O/25:0;O in males. Little is known about the potential role of these metabolites in the brain, and thus, these remain an area for further study.

In females, the percent of the time in the center of the open field negatively correlated with methylsuccinic acid, a metabolite downstream of isoleucine metabolism. The percentage of alternation triplets in the Y-maze negatively correlated with 3-methylpyrazole in females. Little is known about the role of methylsuccinic acid and 3-methylpyrazole in the brain; thus, these metabolites remain an area for further investigation. PC25:1, a phosphatidyl choline, negatively correlated with the percent of time spent in the target quadrant of the MWM in females. This phosphatidylcholine was reduced by obesity in females. A loss of choline-containing phospholipids can contribute to neurodegenerative diseases, and reduced levels are associated with increased AD pathology [60,61]. Thus, decreased levels of brain phosphatidylcholines in obesity may be an additional mechanism by which obesity contributes to worsened cognitive function in females.

### 3.5. Comparison to Data from Other Obese Murine Models

We sought to compare our findings to work performed in other genetically obese murine models to form a more comprehensive view of the effects of obesity on the brain metabolome. Unfortunately, there are not many studies to be found. The studies that we were able to find were conducted in the *db/db* model and were aimed at addressing the effects of T2DM on the brain metabolome, likely utilizing this model due to its more severe T2DM phenotype [28]. We previously conducted a study, similar to the current one, in the *db/db* model to understand the effects of T2DM on the brain metabolome. While there were differences in the brain metabolic response to the *db/db* and *ob/ob* models, there were also similarities. For example, the *db/db* model exhibited a greater overall effect on the brain metabolome in males as compared to females and a female-specific alteration in neuro-modulatory metabolites [40]. Other studies in *db/db* mice addressing brain metabolism were conducted in male mice only and mostly on dissected brain regions rather than whole brain tissue; thus, comparisons to our data were difficult [62,63,64]. Of note, one study did address whole brain tissue and found a reduction in NAD+ levels in *db/db* mice compared to their WT counterparts [64], supporting our results indicating that obesity has an effect on NAD+ metabolism. The study of the brain metabolome in both sexes of other obese murine models remains an area for further study. It may help contribute to our overall knowledge of the effects of obesity on the brain metabolome and tease apart the contributions of obesity from those of inevitable confounding variables found in each model.

### 3.6. Study Limitations

As pointed out earlier, there are multiple mouse models of obesity; we chose the widely used and well-characterized *ob/ob* model, which exhibits a phenotype of obesity, glucose intolerance, and insulin resistance. Although the effects of obesity are difficult to parse out from those of a T2DM phenotype that is present in the *ob/ob* model, obesity in humans is also often accompanied by T2DM. Therefore, the findings using this model are still relevant to human obesity. While we were able to identify some significantly over-represented pathways from the differential metabolites, only a small number of metabolites mapped to pathways. Further, not all metabolites had recognized identifiers, and thus, some metabolic pathways affected by obesity may not be captured.

## 4. Materials and Methods

### 4.1. Experimental Animals

Research was conducted in conformity with the Public Health Service Policy on Humane Care and Use of Laboratory Animals, and reporting was performed in compliance with the ARRIVE guidelines [65]. The institutional review board of the University of California, Davis, the Institutional Animal Care and Use Committee, approved a protocol detailing the research question, all procedures, and planned analyses (protocol number 22598). Food and water intake, as well as activity, was monitored daily by vivarium staff to ensure the well-being of the animals. All procedures were conducted to reduce any discomfort to the mice, and euthanasia was established as a humane endpoint. To minimize confounders, experimental procedures were carried out in the same order and at the same time points relative to mouse age.

Some studies indicate that *ob/ob* mice may develop memory deficits between 13 and 23 weeks of age [66,67,68,69]. Therefore, we chose to study mice between 15 and 18 weeks of age to fall within this time frame. Male and female *ob/ob* mice (stock number 000632 and strain B6.Cg-Lep<ob>/J, Jackson Laboratories, Bar Harbor, ME, USA) and WT mice of the C57Bl/6J strain (Jackson Laboratories, stock 000664) were used for these studies. There were four study groups (n = 20 per group): male *ob/ob*, female *ob/ob*, male WT, and female WT. Mice were housed individually in duplex cages in a temperature- and humidity-controlled environment with a 12 h light/dark cycle in the University of California, Davis Mouse Biology Program. The mice were fed the AIN-93M purified diet (catalog number, TD.00102 Envigo Teklad diets, Madison, WI, USA) ad libitum throughout the study. This standard diet is composed of 4.1% fat, 68.3% carbohydrate, and 12.4% protein (*w*/*w*).

**Figure 7:**
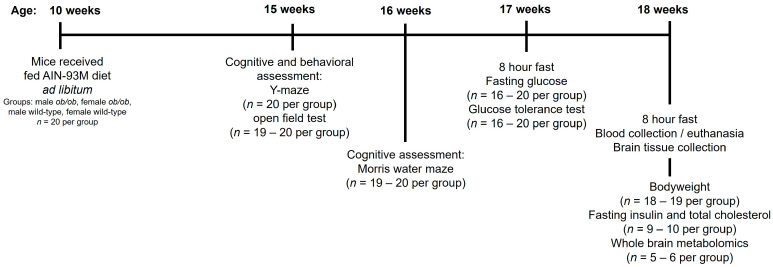
Experimental timeline and endpoints.

Mice were fasted for eight hours prior to euthanasia by exsanguination via ventricular puncture under a combination of ketamine and xylazine anesthesia. Whole brain tissue was quickly collected for assessment of brain metabolomics. Euthanasia was conducted at least one hour after transport to the investigator’s laboratory to ensure the mice had acclimated and minimize the impact of transportation. Cognitive and metabolic testing were conducted in the weeks leading up to euthanasia. An experimental timeline, shown in Figure 7, details each assessment and the number of animals or samples used for each assessment. Details of the procedures performed are provided in the following sections. For all endpoints, the experimental unit was one mouse or a sample obtained from one mouse.

### 4.2. Peripheral Metabolic Testing for Glucose, Glucose Tolerance, Insulin, and Total Cholesterol

Fasting glucose and glucose tolerance were assessed a week prior to euthanasia. After an 8 h fast, blood was collected (approximately 5 µL) from a tail tip cut onto a glucose test strip. Following the initial fasted blood sampling, a GTT was performed by intraperitoneally injecting a 20% glucose solution at a dose of 2 g/kg body weight. Blood samples for determination of glucose levels were then taken through the initial tail tip cut onto glucose test strips at 15, 30, 60, and 120 min after glucose injection. Blood glucose from all samples was determined using Accu-Chek Aivia meter with Accu-Chek Aivia plus test strips (Roche, Basel, Switzerland).

Fasting blood samples for measurement of fasting insulin and total cholesterol were obtained by ventricular puncture under anesthesia at the time of euthanasia. Serum was separated from whole blood by centrifugation and stored at −80 °C until assayed. Total cholesterol was measured using enzymatic assays from Fisher Diagnostics (Middleton, VA, USA). Insulin was determined by electrochemiluminescence from Meso Scale Discovery (Rockville, MD, USA). Both assays were performed in triplicate by the UC Davis Mouse Metabolic Phenotyping Center.

### 4.3. Behavioral and Cognitive Testing

#### 4.3.1. Y-Maze

The Y-maze is widely used to assess spatial and learning memory and active retrograde working memory [70]. The test records the behavior of mice placed in a Y-shaped maze based on how often they explore alternating arms of the maze [71]. Mice were placed in the Y-shaped maze with three white plastic arms (40 cm at 120° angles) facing a random corner. After being placed in the center of the maze, the mice were allowed to freely explore the three arms of the Y-maze for 8 min. A video camera was used to record the number of entries into each arm.

#### 4.3.2. Open Field Test

The open-field test assesses locomotor activity and anxiety-like behavior in mice [57]. The test consisted of placing mice in the corner of a Plexiglas test arena (60 × 40 cm) and allowing them to explore for 20 min. A video camera was used to record their movement. Distance traveled and percent of time spent in center were determined.

#### 4.3.3. Morris Water Maze (MWM) Test

The MWM test is widely used to evaluate spatial learning and memory in rodents [72]. The apparatus consists of a water-filled pool with a hidden escape platform beneath the surface of the water. When the animal is released into the water, it will swim around the pool to look for a platform to escape from the water. The spatial memory is separately assessed in a probe trial without the platform. The version of the MWM used in this study is a modification of the original version of this test originally designed to study spatial memory and learning in rats [73], as was described previously [71].

Mice were placed in a pool with a diameter of 100 cm and temperature of 21 ± 1 °C. The procedure consisted of four days of training, followed by a probe test on the fifth day. The first trial was performed with a visible platform one centimeter above the water level in the pool. Mice that failed to reach the platform on three visible trials were eliminated from the testing. Subsequent test trials were conducted with a hidden platform one centimeter below the water level in the pool. The mice were then subjected to four trial sessions daily, with different starting locations; the maximum trial duration was 90 s. For the probe trial (day 5), the platform was removed, and each animal was allowed to explore the arena for 90 s. Animal behavior was automatically tracked using the SMART 3.003 program. For training sessions, path length to target was used as a measure of learning. For the probe trial, mice were observed for the tendency to explore near the site of the platform in the training sessions. The distance traveled in the target quadrant relative to total distance traveled on the probe trial was used to assess performance.

### 4.4. Metabolomics

The whole brain was collected as quickly as possible following euthanasia, snap-frozen in liquid nitrogen, and stored at −80 °C. The tissue was then powdered, while being kept frozen (all tools and samples were kept on dry ice). Powdered tissue was stored at −80 °C (or on dry ice during transport) until analyses were completed.

Untargeted metabolomics analyses were performed by LC-MS at Creative Proteomics as described previously [40]. Normalization was conducted based on the total ion count method [74]. Details of the untargeted metabolomics methodology can be found in the Supplemental Methods. Although blinding was not possible for most of the study due to obvious phenotypic differences between the *ob/ob* and WT mice, Creative Proteomics was blinded as to the group allocation of the brain samples they received.

### 4.5. Metabolite Pathway Analysis

We obtained the Kyoto Encyclopedia of Genes and Genomes (KEGG) identifier for each of the metabolites which have one. Using these identifiers, we conducted pathway over-representation analyses of the significantly changed metabolites utilizing the IMPaLA software, version 13 [75,76,77].

### 4.6. Statistical Analysis

For body weight, GTT, serum parameters, and behavioral/cognitive testing, outliers were identified using ROUT (Q-1%) outlier test and removed. One outlier data point was removed for insulin levels in the *ob/ob* female group, two male *ob/ob* data points were removed from the Y-maze percent alternation triplets, and one female WT data point was removed for MWM distance traveled. If the data were normally distributed as determined by Kolmogorov–Smirnov test, groups were compared by *t*-test (with Welch’s correction if equal variance assumption was not met). For data that were not normally distributed, Kolmogorov–Smirnov test was used. These analyses were conducted using GraphPad Prism 10 statistical software (GraphPad Software, San Diego, CA, USA).

Analysis of the untargeted metabolomics data, including PLS-DA, pairwise comparisons, generation of volcano plots, and hierarchical clustering of all measured metabolites, was conducted using MetaboAnalyst 5.0 [78,79]. Normalized peak intensity data were provided by Creative Proteomics. No filtering was applied, and data were auto-scaled prior to analyses in MetaboAnalyst. Hierarchical clustering heatmaps of all measured metabolites were generated using Pearson as a distance measure and Ward clustering method. For pairwise comparisons, parametric t-tests were used, and differences were considered statistically significant if adjusted *p* < 0.05. Venn diagrams were generated using BioVenn [80]. Heatmaps of specific subsets of metabolites were conducted using Morpheus [81] with one minus Pearson correlation metric and average linkage method.

Obesity-induced delta correlation analyses of metabolites with peripheral metabolic characteristics, behavioral analyses, and cognitive analyses were conducted as follows: The delta value for each *ob/ob* mouse was determined by subtracting the average of the values obtained for the WT mice of the same sex. Pearson’s correlation analyses were then conducted for each of the metabolites identified as significant between *ob/ob* and WT for each sex. Correlation analyses were performed, and matrix figures were generated using SRplot [82,83].

## 5. Conclusions

**Figure 8:**
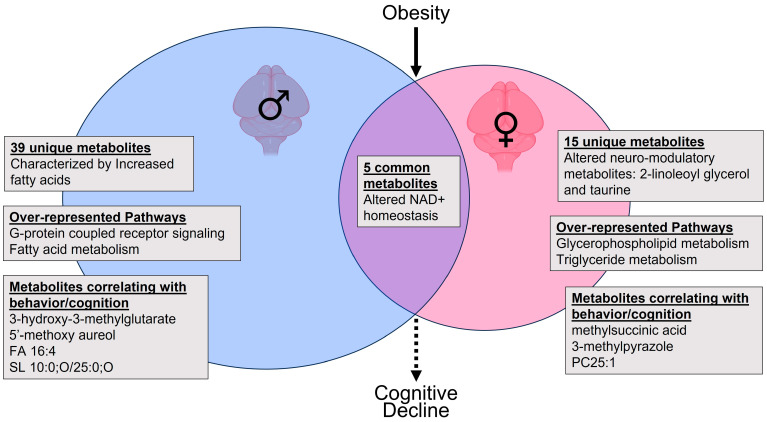
Conceptual summary of the impact of obesity and sex on the metabolomics of the murine brain.

In conclusion, we found that obesity modifies the murine brain metabolome with sex-dependent and -independent effects. We conceptualize and summarize our findings and how they may have implications for cognitive decline in Figure 8. Obesity had a stronger effect on the brain metabolome than biological sex. However, most metabolites altered by obesity were sex-specific and predominated in males. In contrast, in females, obesity affected behavior and neuro-modulatory metabolites. In both sexes, obesity altered NAD+ homeostasis in the brain. In addition to most obesity-altered metabolites being unique to one of the sexes, the common metabolites demonstrated different relationships to phenotypic measures in males and females. Thus, biological sex, in addition to being an important modifier of the brain metabolome itself, modifies the relationship of brain metabolites to behavioral and cognitive phenotypes. Our work highlights the complexity and importance of addressing the interaction between biological sex and obesity and its impact on brain metabolome. As such, our findings have potential implications for sex-specific approaches to therapeutics for the rising and dual epidemics of obesity and dementia.

## Figures and Tables

**Table 1:** Body weight and peripheral metabolic characteristics.

	Males		Females	
	WT	*ob/ob*	WT	*ob/ob*
Body weight (g)	29.5 ± 2.7	51.5 ± 3.3 *	20.6 ± 1.4	52.4 ± 4.2 *
Glucose (mg/dL)	157.4 ± 32.6	175.6 ± 41.6	126.3 ± 18.5	177.7 ± 54.3 *
GTT AUC (mg * min/dL)	29,500 ± 5380	43,307 ± 8706 *	23,323 ± 2106	47,047 ± 8270 *
Insulin (pg/mL)	229.1 ± 131.7	3311.4 ± 1625.2 *	182.2 ± 78.7	3769.2 ± 729.6 *
Total cholesterol (mg/dL)	124.1 ± 35.9	279.1 ± 25.9 *	77.0 ± 17.8	290.5 ± 15.9 *

Data shown are mean ± standard deviation. * *p* < 0.05 compared to sex-matched WT mice. GTT AUC: glucose tolerance test area under the curve.

## Data Availability

Data summaries are available in the Supplemental Materials. Raw data will be provided upon request.

## Supplementary Materials

The following supporting information can be downloaded at: https://www.mdpi.com/article/10.3390/ijms25063475/s1.
