# Supplementary material for: The Brain Metabolome Is Modified by Obesity in a Sex-Dependent Manner
Source: Int J Mol Sci. 2024 Mar 20;25(6):3475. doi: 10.3390/ijms25063475 (PMC10970387; doi:10.3390/ijms25063475)
Supplement: Supplementary file 1 [file ijms-25-03475-s001.zip › ijms-2905830-supplementary.pdf]

Supplementary Materials for “The brain metabolome is modified by obesity in a sex-dependent manner”

Supplemental Methods: Untargeted metabolomics details

Untargeted metabolomics was performed as follows: Fifty milligrams of tissue was combined with 800 µL of 80% methanol in a tube. The mixture was vortexed for 30 seconds, homogenized for 90 seconds by adding two 5-mm metal balls to the tube and using a MM 400 mill mixer at 30 Hz, then sonicated for 30 minutes at 4°C. Following this, samples were kept for one hour at -20°C, vortexed for 30 seconds, and kept at 4°C for 30 minutes. After centrifugation, 200 µL of supernatant was combined with 5 µL of 0.5 mg/mL DL-o-Chlorophenylalanine and transferred to a vial for LC-MS analysis.

The LC-MS system consisted of ACQUITY UPLC HSS T3 (100×2.1mm×1.8 µm) with ACQUITY UPLC (Waters, Milford, MA, USA) combined with Q Exactive MS (Thermo Fisher Scientific, Waltham, MA, USA) and screened with electrospray ionization (ESI) mass spectrometry. The mobile phase was composed of solvent A (0.05% formic acid water) and solvent B (acetonitrile) with a gradient elution (0-1 min, 5% B; 1-12 min, 5%-95% B; 12-13.5 min, 95% B; 13.5-13.6 min, 95%-5% B; 13.6-16 min, 5% B). The flow rate of the mobile phase was 0.3 $mL \cdot min^{-1}$. The column temperature was maintained at 40°C, and the sample manager temperature was set at 4°C. Mass spectrometry parameters are shown in Table S1.

The same amount of extract was obtained from each sample and mixed as quality control (QC) samples. The QC sample was prepared using the same sample preparation procedure. DL-o-Chlorophenylalanine was used as internal standard in this assay.

The resulting peak areas were normalized for each ESI mode separately, utilizing the following equation: normalized data (peak 1) = raw data (peak 1)/sample (total peak area)*1000000. This calculation is based on the total ion count method, the most common and simple approach to normalization of LC-MS data [1].

1. Nezami Ranjbar, M.R.; Zhao, Y.; Tadesse, M.G.; Wang, Y.; Ressom, H.W. Evaluation of Normalization Methods for Analysis of LC-MS Data.; 2012; pp. 610–617.

Supplemental Tables

Table S1. Mass spectrometry parameters

**ESI mode**	**Positive**	**Negative**
Heater temperature (°C)	300	300
Sheath gas flow rate (arb)	45	45
Auxillary gas flow rate (arb)	15	15
Sweep gas flow rate (arb)	1	1
Spray voltage (kV)	3.0	3.2
Capillary temperature (°C)	350	350
S-Lens radiofrequency level (%)	30	60

ESI (electrospray ionization)

Table S2. Metabolites significantly altered by obesity in males

Metabolite	ESI Mode	RT (min)	m/z	FC	log2(FC)	adjusted p-value
Glycerol 3-phosphate	+	11.14	173.0211	0.41848	-1.2568	0.000117
Aniline	+	0.092	94.06563	0.69116	-0.5329	0.000646
Nicotine	+	9.099	163.1228	0.71229	-0.48947	0.014673
PC 20:2e; PC 18:2e/2:0	+	10.684	548.3705	0.71233	-0.48937	0.032779
Orcinol	+	0.014	125.06	0.74979	-0.41544	0.01397
Iditol	-	8.796	181.0705	0.78453	-0.3501	0.017802
2-Hydroxyvaleric acid	-	2.999	117.0543	0.79769	-0.3261	0.041682
Dulcitol	-	14.721	181.0706	0.79933	-0.32314	0.041682
Hexylamine	+	8.498	102.1281	0.8105	-0.30311	0.047846
Diacetyl	+	0.045	87.04469	0.81948	-0.28723	0.039902
3-Hydroxy-3-methylglutarate	-	6.518	161.0443	0.82053	-0.28537	0.032779
Allose	-	6.071	161.0443	0.82888	-0.27076	0.0025
Phthalic acid	-	6.413	165.0181	0.84465	-0.24357	0.025113
2-Deoxyglucose	-	0.09	163.0598	0.84908	-0.23602	0.041682
Mandelic acid	-	6.93	151.0388	0.86019	-0.21727	0.043999
2-Oxobutyric acid	-	1.225	101.0229	0.89799	-0.15524	0.043999
Ticagrelor	+	10.658	523.191	0.90295	-0.14729	0.041682
Myristic acid	-	12.435	227.2009	1.2448	0.31597	0.034213
Isovaleric acid	+	2.849	103.0758	1.3041	0.38308	0.041682
4-Hydroxyphenyllactic acid	-	3.4	181.0496	1.3672	0.45126	0.025717
Ergocryptine	+	10.217	534.2951	1.3899	0.47493	0.037296
Ethosuximide	+	2.853	142.0863	1.39	0.47503	0.017802
4-Methoxyaniline	+	2.852	124.0759	1.3939	0.4791	0.011467
Roccellic acid	-	12.665	299.2222	1.4152	0.50103	0.017802
Epinephrine	+	2.854	184.0969	1.4229	0.50883	0.032779
Pantothenic acid	-	2.84	218.1025	1.4326	0.51867	0.039902
Palmitoleic acid	-	12.666	253.2167	1.4432	0.5293	0.006879
Linoleic acid	-	12.929	279.2324	1.4537	0.5397	0.007078
Sarcosine	+	2.85	90.05552	1.4577	0.54365	0.023725
Betonicine	+	2.853	160.0969	1.4833	0.56876	0.032779
2-Hydroxy-6-pentadecylbenzoic acid	-	11.464	347.2585	1.5002	0.58516	0.039902
Nudifloramide	+	1.666	153.0658	1.6072	0.68459	0.043999
FA 16:4	-	10.874	247.1697	1.6196	0.69567	0.04816
LysoPC(0:0/18:0)	+	11.543	562.3264	1.6336	0.70802	0.014673
FA 16:3	-	11.557	249.1854	1.768	0.82211	0.014108
PS 36:2	+	14.501	788.5415	2.1288	1.09	0.017802
Xanthohumol	-	12.666	353.1421	2.3579	1.2375	0.039902
Vanillin-4-sulfate	-	4.702	230.9962	3.207	1.6812	0.002555
Corticosterone	-	7.107	391.2122	3.2281	1.6907	0.003362

Linolenic Acid	-	12.298	277.2168	3.4328	1.7794	0.001217
5'-Methoxy aureole	+	7.126	329.2107	4.0159	2.0057	0.000567
SL 10:0;O/25:0;O	+	14.502	651.533	5.8273	2.5428	0.003362
NAGly 10:0/10:0	+	11.922	417.3358	17.498	4.1291	0.01238
PS 20:3_20:3	+	14.503	836.5398	50.308	5.6527	0.000567

ESI (Electrospray ionization), RT (retention time), m/z (mass to charge ratio), FC (fold change)

Table S3. Metabolites significantly altered by obesity in females

Metabolite	ESI Mode	RT (min)	m/z	FC	log2(FC)	adjusted p-value
2-Linoleoyl glycerol	+	14.497	337.2734	0.12592	-2.9894	0.034955
Glycerol 3-phosphate	+	11.14	173.0211	0.37325	-1.4218	0.04146
2-Aminoadipic acid	-	0.795	160.0602	0.41656	-1.2634	0.00828
Bentazone	-	7.224	239.0474	0.48132	-1.0549	0.04146
Methylsuccinic acid	-	2.194	131.0336	0.51213	-0.96541	0.005157
2-phenylphenate tetrahydrate	+	4.206	171.0803	0.61288	-0.70633	0.005157
3-Hydroxy-3-methylglutaric acid	-	1.597	161.0443	0.64851	-0.6248	0.025786
PC 25:1	+	7.363	656.4324	0.82762	-0.27296	0.024513
3-Hydroxy-3-methylglutarate	-	6.518	161.0443	0.83205	-0.26525	0.027369
Tropine	+	5.94	142.1226	1.1484	0.19957	0.019474
Taurine	-	0.768	124.0059	1.2288	0.29727	0.019474
3-Methylpyrazole	+	0.782	83.06102	1.3142	0.39417	0.005157
4,4'-Sulfonyldiphenol	-	0.769	249.0213	1.4011	0.48656	0.041323
4-Hydroxybenzotriazole	+	0.669	136.0506	1.9268	0.94619	0.025786
Xanthohumol	-	12.666	353.1421	1.9304	0.94891	0.024513
Nudifloramide	+	1.666	153.0658	2.0664	1.0471	0.031958
Vanillin-4-sulfate	-	4.702	230.9962	2.076	1.0538	0.019474
albocycline	-	14.504	307.1945	11.175	3.4823	0.024513
3-(3-Hydroxyphenyl)propionic acid sulfate	-	4.034	245.0121	24.557	4.618	0.025786
2,3-dinor Prostaglandin E1	-	14.105	307.1946	71.279	6.1554	0.013721

ESI (Electrospray ionization), RT (retention time), m/z (mass to charge ratio), FC (fold change)

Table S4. Significant correlations between obesity-altered brain metabolites and phenotypic measures in males

Phenotypic measure	Significant correlations with Metabolites
**Peripheral metabolic characteristics**	
**Total Cholesterol**	None
**Insulin**	4-Methoxyaniline, p=0.0258, R=-0.8657<br>Betonicine, p=0.0371, R=-0.8384<br>Ethosuximide, p=0.0327, R=-0.8485<br>Isovaleric acid, p=0.0333, R=-0.8472<br>Linoleic acid, p=0.0240, R=0.8708<br>Sarcosine, p=0.0059, R=-0.9364
**Glucose**	4-Hydroxyphenyllactic acid, p=0.0240, R=-0.8708<br>Betonicine, p=0.0341, R=0.8451<br>Epinephrine, p=0.0456, R=0.8202<br>FA 16:3, p=0.0293, R=0.8567<br>Nicotine, p=0.0275, R=0.8613<br>Phthalic acid, p=0.0386, R=0.8349
**GTT AUC**	5'-Methoxy aureol, p=0.0285, R=-0.8587<br>SL 10:0;O/25:0;O, p=0.0351, R=0.8428
**Behavioral and cognitive tests**	
**Open field distance**	Corticosterone, p=0.0118, R=-0.9100
**Y-Maze distance**	None
**MWM distance**	Hexylamine, p=0.0128, R=0.9062<br>Linolenic acid, p=0.0170, R=0.8917
**Open field % time in center**	3-Hydroxy-3-methylglutarate, p=0.0089, R=0.9222
**Y-maze % alternation triplet**	None
**MWM % distance in target quadrant**	5'-Methoxy aureol, p=0.0140, R=-0.9019<br>FA 16:4, p=0.0279, R=0.8603<br>SL 10:0;O/25:0;O, p=0.0352, R=0.8426

Table S5. Significant correlations between obesity-altered brain metabolites and phenotypic measures in females

Phenotypic measure	Significant correlations with Metabolites
**Peripheral metabolic characteristics**	
**Total Cholesterol**	3-(3-Hydroxyphenyl)propionic acid sulfate, p=0.0463, R=0.8845<br>3-Hydroxy-3-methylglutaric acid, p=0.0016, R=-0.9879<br>Xanthohumol, p=0.0325, R=-0.9090
**Insulin**	Glycerol 3-phosphate, p=0.0090, R=-0.9617
**Glucose**	None
**GTT AUC**	None
**Behavioral and cognitive tests**	
**Open field distance**	2-Linoleoyl glycerol, p=0.0021, R=-0.9856
**Y-Maze distance**	None
**MWM distance**	Taurine, p=0.0395, R=-0.8962
**Open field % time in center**	Methylsuccinic acid, p=0.0093, R=-0.9606
**Y-maze % alternation triplet**	3-Methylpyrazole, p=0.0150, R=-0.9459
**MWM % distance in target quadrant**	PC 25:1, p=0.0110, R=-0.9559
